# Non-communicable Diseases Among Tribal Populations in India: Epidemiology, Social Determinants, and Tailored Public Health Approaches

**DOI:** 10.7759/cureus.96899

**Published:** 2025-11-15

**Authors:** Swathika Devi R, Anantha Eashwar V M, Sujitha Pandian, Monica Albert Sekhar, Ponmalar Manivannan

**Affiliations:** 1 Community Medicine, Sree Balaji Medical College and Hospital, Chennai, IND

**Keywords:** epidemiological transition, india, non-communicable diseases, public health intervention, social determinants, tribal health

## Abstract

Non-communicable diseases (NCDs) have become a major public health concern among India’s tribal populations, who face unique vulnerabilities. Historically burdened by infectious diseases and malnutrition, tribal communities are now undergoing a rapid epidemiological transition, with rising prevalence of cardiovascular diseases, diabetes, hypertension, and cancers. Behavioral risk factors such as tobacco and alcohol use, poor nutrition, and declining traditional food practices intersect with socio-economic disadvantages, low health literacy, and limited healthcare access to exacerbate this burden. Women and Particularly Vulnerable Tribal Groups (PVTGs) are disproportionately affected. Despite national policies and tribal health initiatives, barriers, including cultural mismatch of interventions, infrastructure gaps, and poverty, persist. This narrative review highlights epidemiological evidence, social determinants, and systemic barriers shaping NCD outcomes among Indian tribes. It further emphasizes the need for culturally sensitive, community-driven, and equity-focused strategies to strengthen prevention, screening, and management, ultimately reducing health disparities.

## Introduction and background

Non-communicable diseases (NCDs) such as cardiovascular diseases, diabetes mellitus, high blood pressure/hypertension, and cancer have emerged as the leading causes of adult deaths in India, including among various tribal communities. With over 104 million people legally recognized as Scheduled Tribes, tribal communities represent a unique and vulnerable population group with unique socio-cultural practices, remote living conditions, and chronic socio-economic marginalisation [1]. Recent scholarship indicates that tribal communities have been undergoing a rapid health transition, from a dominating presence of infectious diseases and nutritional disorders to a rising NCD burden - a transition spurred by poverty, low literacy levels, low information about health issues, and limited access to necessary health services [1].

Prevalence patterns among tribal communities also differ from those of the general population because of variations in behavioral norms, environmental exposure, and biological susceptibilities. Higher prevalence of tobacco use and consumption of alcoholic beverages, lower levels of nutrition, a less active lifestyle, and unique genetic profiles contribute to higher susceptibility. Prevalence information for NCDs and management practices remains scarce and inconclusive among tribal communities, and health promotion interventions often turn out to be mismatched to tribal population requirements as well as to tribal values [2].

Since tribal regions experience a dual challenge from chronic infectious diseases and rising NCDs, a comprehensive review and analysis is called for. In this paper, we present a review of the current situation concerning NCD risk factors among Indian tribal groups, explore epidemiological profiles, behavioral and biological determinants, socio-demographic effects, and prevailing strategies for interventions, and introduce culturally sensitive and equitable public health interventions.

Methodology

An extensive internet database search of PubMed, Scopus, and Web of Science was conducted to perform the narrative review. All the articles relevant to the topic were searched for with the keywords "Non-communicable diseases," social determinants," "Tribal population," "Cardiovascular diseases," "Diabetes Mellitus," "Metabolic disorders", Cancer," "Tobacco consumption, Alcohol consumption", Dietary practices," Socio-Cultural dynamics", "Poverty", "National programes for Tribal Health". The inclusion criteria were articles that were published in peer-reviewed journals, English language articles, and on NCDs and levels of prevention. Articles that were published between 2000 and 2024 were considered in the study. In addition, the authors performed manual searches to include other relevant studies. Original research articles, systematic reviews, meta-analyses, and RCTs were selected for inclusion. The articles that had based their study on research that was carried out on the burden of NCDs in India's tribal population and the determinants contributing to social and environmental factors, and the public health measures were considered for inclusion in this narrative review. The rest were excluded. The exclusion factors were any duplicate papers, papers published in non-peer-reviewed journals or in a language other than English, and those with incomplete texts. Articles fitting into the inclusion factors were only selected and screened for inclusion. The authors agreed by consensus to settle any disagreements regarding selecting articles based on the inclusion factors.

## Review

Global and national overview of the tribal population

Indigenous and tribal people are among the most culturally diverse but marginalized population groups in the world. The World Health Organization's Global Plan of Action for the Health of Indigenous Peoples estimates that there are 476.6 million Indigenous people worldwide, making up more than 6% of the world's population [3]. This diversity is spread across more than 90 nations with over 5,000 different Indigenous and tribal groups, with the highest concentration in Asia at 70.5% of the world's total Indigenous population. Indigenous people with such significant cultural contributions are plagued by disproportionate socioeconomic problems everywhere. According to the World Bank, although they comprise only 6.2% of the world's population, they represent 18.2% of the global population living in extreme poverty [4]. Their life expectancy is calculated to be as much as 20 years lower than that of non-Indigenous people, and they are confronted with obstructions associated with land loss, discrimination, and institutional exclusion [3]. A large worldwide study by Anderson et al., published in *The Lancet *and implemented through the Lancet-Lowitja Institute Global Collaboration, examined Indigenous health in 23 countries spanning all WHO global regions. Their results showed that Indigenous groups routinely show worse health and social outcomes than their respective benchmark populations, although this pattern is not consistently seen for all indicators [5].

African Indigenous communities, while constituting 11.5% of the world’s total Indigenous population, possess extraordinary cultural and language diversity and unique challenges. Ohenjo et al.'s systematic review in *The Lancet *of Indigenous African health documented that Indigenous communities face multi-dimensional health concerns, with obstacles varying from impaired access to care, poor infrastructure, institutionalized discrimination, and displacement. Contemporary research documents ongoing shifts in African Indigenous communities [6].

India has the world's second-largest population of Indigenous people, with 705 Scheduled Tribes officially recognized and covering approximately 104.3 million people, or 8.6% of the country's population [7]. Demographic diversity is associated with phenomenal cultural, linguistic, and health differences between tribes. Ganesan** **et al.'s extensive review in the *Indian Journal of Public Health *reported that healthcare, education, and economic opportunities are disproportionately hindered for the Indian tribal population. The review confirmed that approximately 2.6 million people are part of the Particularly Vulnerable Tribal Groups (PVTGs), the most marginalized populations [8]. The distribution of the Indian tribal population is characterized by clear geographical clustering. Madhya Pradesh has the highest population percentage (14.7%), followed by Maharashtra (10.1%) and Odisha (9.2%). Other notable concentrations were Rajasthan (8.9%), Gujarat (8.6%), Jharkhand (8.3%), and Chhattisgarh (7.5%). Northeastern regions such as Mizoram, Nagaland, and Meghalaya have tribal populations of over 30% of their entire population [9].

NCDs among tribal populations

Around 66% of all deaths in the tribal districts may be attributed to non-communicable diseases, which have now emerged as an important health problem in the tribal districts. The situation has now morphed from an epidemiological transition characterized by communicable disease dominance to a new dominance of non-communicable diseases among them [10].

Cardiovascular Diseases

Cardiovascular diseases are one of the major contributors to NCD-related mortality among the tribal population, accounting for 24% of all deaths and serving as the leading cause of mortality in 75% of the tribal districts in India [1]. Approximately 29% of tribal adults suffer from hypertension, while almost 60% of the cases remain undiagnosed due to gaps in screening and healthcare access. Regional studies increasingly support this trend. Kandpal et al documented a prevalence of hypertension among adults aged >30 years at 32.6% in the Tharu tribe from Uttarakhand [11]. Babu et al, a multicenter study, reported a diabetes prevalence of 4.77% in the tribes from six districts of India [12]. In Northeast India, some tribal populations living in Mokokchung, Nagaland, exhibit higher prevalence levels. Tushi et al reported that almost half of male respondents (58.9%) and a similar proportion (49.6%) of women were tobacco users in smokeless form, and hypertension was as high as 43.2% [13]. In South India, Ponruban et al have examined tribal groups in Jawadhu hills, Vellore, which has shown that the hypertension rate was 17.7%[14].

Diabetes and Metabolic Disorders

Studies indicate that the prevalence of diabetes among the tribal population ranges from 3.7% to 10.2%. Impaired fasting glucose affects approximately 5.1% to 13.5% of the tribal population, and impaired glucose tolerance is found among 6.6% to 12.9% of the tribal population. This indicates a substantial population of individuals who are prediabetic and at risk of developing a full-blown disease [2]. During routine diabetic screening, approximately 77% of type 2 diabetes mellitus (T2DM) cases are newly diagnosed, highlighting the burden of the disease in these areas [15]. Studies among tribal populations in Kerala showed that diabetes rates were between 3.9% and 11.7% [16].

Cancer Burden

Cancer is the second major cause of death related to NCDs among the tribal population, accounting for approximately 13% of the total mortality. This burden exhibits dramatic variation, with a higher prevalence among tribes in the northeastern region than among other tribes. This could be attributed to the higher use of alcohol and tobacco in these tribal areas [17].

This growing vulnerability to NCDs among tribal peoples is determined by a range of intersecting systemic and sociocultural issues, including inadequate availability of healthcare facilities, low health literacy and awareness, economic deprivation, barriers to comprehensive care arising from language and cultural differences in service provision, and an evidenced reluctance or inability to conduct health research on tribal peoples (as a population compared to non-tribal peoples). For this reason, tribal peoples are essentially experiencing a double burden of disease, as they need to contend with established historical public health issues along with the increasing challenge of chronic lifestyle-associated diseases [18].

Risk factors of NCDs among tribal populations

Tobacco Use

Among the tribal populations. Tobacco use is the most common behavioral risk factor for NCDs, with almost 72% to 80% of communities indulging in tobacco use in one form or another. It is increasingly used among males and older adults. There is equally troubling evidence about the PVTGs, such as the Baiga tribe of Madhya Pradesh. Chakma et al. found the prevalence of hypertension, diabetes, and tobacco use to be 27.6%, 5.7%, and 73.4%, respectively [12], which in turn are associated with various cardiovascular diseases, hypertension, and cancers [19-21].

Alcohol Consumption

Alcohol consumption is increasingly high among the tribal population, with approximately 60% to 80% of the population engaging in the practice. Regular and binge drinking patterns were more common among men and older adults. Many tribal communities consume home-brewed traditional alcohol, and the integration of these alcohol habits as part of the community aspects has shown use rates between 40% and 70% among adult men for tribes in various studies [22]. This could indirectly lead to an increased prevalence of liver disease, hypertension, and diabetes within these communities. The barriers to addressing this include alcohol being accepted as a part of social practice and a lack of health awareness regarding the ill effects of alcohol consumption [23].

Unhealthy Dietary Practices

Since people living in the tribal areas have limited access to resources, they are often prone to limited access to the necessary intake of fruits and vegetables, with surveys indicating as high as 98% of the tribal population having low dietary diversity. The decline in the consumption of traditional produce and grains has led to an increase in the burden of diabetes and obesity. A balanced diet in a tribal community rests on centuries of evolution to suffice nutritionally because of ethnic traditional food systems. All tribal diets around the world show a stunning equilibrium through the inclusion of several food groups, including wild plants and animals, cereals, and other products from the forest, creating ethnically holistic patterns constituting a complete diet that nurtured health and well-being for many generations [24]. Studies have revealed a sharp decline in the consumption of wild meat in tribal societies and its accompanying negative impact on nutritional well-being and socio-cultural life [25]. Studies among tribal populations in different parts of the world show that decreased access to wild meat is associated with increased reliance on processed foods, which brings about a nutritional shift along with adverse health effects. Nutrition transition is observable in the Indian tribal areas. While these communities were once dependent on variable dietary patterns comprising diverse amounts of coarse grains in the form of millets, wild greens, and forest foods, they are shifting towards diets that are high in refined carbohydrates or processed foods. Premagowri et al have documented this transition and its implications for NCD risk [26].

Socio-cultural dynamics

Social determinants of health (SDOH) are important components that play a key role in the health outcomes of individuals. These include conditions with which people are born, live, grow, and age, and the access they have to power, money, and resources. This plays a major role in tribal communities, as health follows a social gradient at all income levels, which implies that when the socioeconomic status of the individual is low, it could lead to poor health outcomes [27]. Tribal communities thrive on subsistence-based economies in which they produce goods for their basic survival needs and not in excess. The distinct socio-cultural beliefs rooted in tradition, which make them indulge in tobacco and alcohol consumption, play a major role in making them more vulnerable to developing NCDs [28].

Socio-Demographic Determinants of NCDs Among the Tribal Population

There is widespread variation in NCD patterns based on sex differences, the main one being the acceptance of lifestyle risk factors for NCDs, such as alcohol consumption and tobacco use among males. Although males may be involved in outdoor strenuous work, which may be physically demanding, it could ultimately lead to stress, which could lead to the development of NCDs. Women are at a higher risk of developing NCDs as they are involved in traditional household work, which could lead to the development of obesity and metabolic risk among the female tribal population [23]. Accessing healthcare by women in most tribal populations is difficult because of barriers to healthcare access, including lower educational status, lack of decision-making within the family, and cultural limitations, which could lead to a delay in the diagnosis and effective management of NCDs. Even the food distribution within tribal households practices the habit of prioritizing men over women, leading to micronutrient deficiency and increasing the risk of developing anaemia or obesity, which could ultimately influence NCD patterns among the female tribal population [29].

Many households in tribal areas live in poor conditions with inadequate housing infrastructure, which could impact their health. Lack of privacy and overcrowding could increase stress and reduce well-being, leading to an increased risk of developing NCDs. The higher prevalence of lower socioeconomic status among the tribal population has been found to have a strong association with higher usage of tobacco and alcohol and lack of physical activity [30].

Barriers to NCD prevention and management

Despite the increasing prevalence of NCDs among tribal populations, various barriers and challenges play a role in affecting the early diagnosis and prevention of these NCDs. These are multifaceted and deeply rooted in sociocultural beliefs, tribal economies, healthcare access, and policy-related factors.

The major factor is the healthcare infrastructure in the tribal areas, which lacks specialized care services and adequate diagnostic facilities that are essential for the effective diagnosis and treatment of NCDs. Remotely placed tribal areas and difficulty in reaching the beneficiaries due to transportation barriers cause difficulty in the provision of essential healthcare services [31].

Even though they are diagnosed with the conditions, lack of health literacy among the tribal population leads to poor understanding of preventive measures, the role of regular health follow-ups, and adherence to treatment medication. Belief in traditional medicine pertaining to individual tribes and misconceptions about NCDs play a role in the lack of engagement of the population with necessary healthcare services [32]. These socio-cultural beliefs have a strong influence on health-seeking behavior, with most of the tribal population placing their trust in traditional healers in their village over biomedical approaches. Fear and stigma associated with these chronic diseases could also lead to delaying treatment by concealing the illness [33].

Poverty is another major limiting factor that hinders the ability to afford healthcare costs, including expenses related to travel, diagnostic tests, and medicines. This leads to poor disease control and complications [34]. Linguistic barriers and a shortage of healthcare workers who are familiar with the local languages and culture of the tribes lead to difficulty in establishing trust and rapport among the tribal population, which reduces the quality, effectiveness, and reach of healthcare services within the tribal population [35].

National programmes for tribal health in India

India has designed detailed policies and initiatives aimed at improving tribal health. According to the National Health Policy 2017, tribal communities are identified as having special needs and should be provided with culturally appropriate healthcare [36]. There is a lacuna in the integration of tribal medicine and the provision of adequate training for healthcare professionals to become competent in addressing cultural barriers [37].

Through the Tribal Sub Plan (TSP), all areas, such as health, are encouraged to give equal attention and resources to tribal development. Specific budgets for tribal health infrastructure, human resources, and service delivery are required by the TSP in the health sector [36]. According to evaluation research, the health sector developed infrastructure, but it was not easy to bring in the human workforce, and the lack of proper utilization of the gram sabha and tribal councils reduced the overall efficacy of the services provided [38].

The National Health Mission (NHM) includes some special benefits for the tribal health sector. Through the NHM, the Tribal Health Plan follows a strategy to boost medical infrastructure among Indigenous populations, train tribal health workers in cultural understanding, and address the unique health problems faced by tribal residents [39]. However, sustaining health workers in tribal areas was low due to challenging living conditions and a lack of proper incentives. There are also insufficient data available to effectively provide need-based interventions through community outreach activities [40].

The use of mobile medical units (MMUs) is a new approach for delivering medical care to remote tribal areas. Research indicates that MMU programs make healthcare more accessible for mothers and children, but few have found that services are available for managing ongoing health problems, especially NCDs [41]. Services catered towards chronic non-communicable diseases are found to be inconsistent or absent in most MMU operations. The non-availability of specialists to cater to complex NCD management among the population is another major setback of MMUs [42].

AYUSH (Ayurveda, Yoga, Unani, Siddha, and Homeopathy) is integrated into tribal health programs to honor traditional healing systems. Researchers have claimed that mixing old and new types of medical care might be beneficial, but reliable assessments of these methods are still rare [43].

Specific National Programmes Catering to Tribal Health in India

Dharti Aaba Janjatiya Gram Utkarsh Abhiyan: The Abhiyan seeks to enhance health parameters in the tribal region by ensuring the saturation of government schemes at the village level, with specific emphasis on 100% coverage of health insurance under Ayushman Bharat and periodic health check-ups through outreach camps. Maternal and child health, immunization, and NCD screening are given special focus. The program fosters health literacy and awareness among tribal communities through Information, Education, and Communication (IEC) activities and the involvement of Accredited Social Health Activist (ASHA) workers. It provides functional health and wellness centers (HWCs) in tribal villages to deliver holistic primary healthcare services [44].

Although village-wide screening of NCDs is prioritized, constant follow-up at regular intervals and integration of secondary or tertiary level NCD care are weak, leading to gaps in referrals and a lack of treatment initiation or management of NCD complications. Many HWCs are understaffed regarding NCD management, resulting in the provision of suboptimal services [44].

Pradhan Mantri Janjatiya Unnat Gram Abhiyan (PMJUGA): The program focuses on the holistic development of approximately 63,000 tribal villages in India and seeks to bridge major developmental gaps in health, nutrition, education, and livelihood. The initiative lays a strong emphasis on infrastructure development, such as roads, schools, and health facilities. Particular attention is paid to enabling tribal youth and women through skill development and jobs. It follows a convergence approach by combining the efforts of various ministries and state governments for effective results. Indicators related to NCDs were not always included in either the core targets or the monitoring dashboards, which risks the under-prioritization of resources relative to either livelihoods or infrastructure [45].

TRIFED (Tribal Cooperative Marketing Development Federation of India): TRIFED operates under the Ministry of Tribal Affairs to facilitate tribal entrepreneurship and market integration in India. It promotes tribal producers and artisans by offering support through schemes such as "TRIBES India" for handicraft and forest produce marketing. TRIFED provides skill development camps and training in value addition and product design. It guarantees a Minimum Support Price (MSP) for Minor Forest Produce under the Van Dhan Yojana, which improves tribal incomes. The scheme also helps in maintaining tribal cultural heritage by promoting traditional arts and crafts [45].

Recommendations for addressing tribal health in relation to NCDs in India

To address the growing burden of risk factors for non-communicable diseases (NCDs) in the tribal population, comprehensive, culturally sensitive prevention strategies are essential.

Primordial Prevention [46]

Elimination of risk factors in early childhood through the promotion of healthy behaviors should be aimed at primordial prevention. Educational programs for behavioral risk factors and NCDs in schools within tribal areas must incorporate culture-specific education that focuses on Indigenous games, food habits, and exercise. Anganwadi centers must be strengthened with nutrition and health promotion aspects, and parents must be taught about a health-friendly home environment. Conservation and promotion of traditional dietary patterns, oral anti-tobacco education, and active participation of tribal leaders in health promotion will favor long-term behavioral changes.

Environmental changes at the community level, such as tobacco-free perimeters around schools, home gardens, and safe public spaces for activities, need to be implemented. Tribal elders, women, and youth must be engaged as health champions through social interventions to promote healthy behavior. Policy support should encompass tribal health bylaws restricting the marketing of tobacco and alcohol and encouraging traditional foods. NCD-centric primary health centers at tribal panchayat levels and making access easier through transportation infrastructure are essential for the field-level enforcement of preventive strategies.

Primary Prevention [40]

Primary prevention addresses the reduction in the prevalence of modifiable risk factors among already vulnerable populations. Community-led cessation programs for tobacco and alcohol should incorporate Indigenous healing processes and marry them with contemporary strategies, assisted by peer educators and schemes in alternative livelihoods. Encouraging physical activity-based traditional occupations such as agriculture and fishing, home-based activities, and seasonal exercise programs will reverse the increasing sedentary lifestyle.

Nutrition education using culturally acceptable foods needs to be provided through tribal outreach programs. Kitchen gardening, traditional cooking modifications, and food preservation training should be prioritized to enhance dietary diversity. Mobile screening units and regular check-ups at primary health centers should be established to facilitate early detection of hypertension, diabetes, and obesity. Community health workers need training to carry out risk assessments, and the inclusion of traditional healers in formal health services must be considered. Building local health structures, supply systems, and referral links is vital for long-term disease prevention.

Expanded Public Health Actions [40]

Culturally Adapted Health Promotion: Establish community-level educational interventions for tobacco, alcohol, poor diet, and physical inactivity through locally appropriate media, traditional arts, and local languages.

Systematic NCD Screening: Systematic screening of blood pressure, blood glucose, and BMI at the primary healthcare level should be implemented to allow early diagnosis and management.

Nutritional Interventions: Encourage food diversification and nutritional literacy via existing tribal health services and integrate this into national outreach programs, such as Rastriya Bal Swasthiya Karyakaram (RBSK) and Integrated Childhood Development Services (ICDS).

Policy Integration: Tribal-specific NCD data must be integrated into national and state-level policies, especially under the National Programme for Prevention and Control of Non-Communicable Diseases (NP-NCD), to aid in context-specific planning.

Table 1 presents a review of literature on various studies highlighting the risk factors and prevalence of NCDs among tribal populations.

**Table 1:** Evidence From Literature on the Risk Profile and Prevalence of NCDs in Tribal Populations NCD: non-communicable disease

S. No.	Author, Location, Year of Study	Results
1	Babu BV et al, 2024, India [12]	The study conducted among the tribal population of India revealed that the prevalence of type 2 diabetes, based on random blood glucose (RBG), was 4.77%, while based on fasting blood glucose (FBG), it was 6.80%. The analysis showed that age, smokeless tobacco use, hypertension, and obesity were significantly associated with the occurrence of type 2 diabetes among the study participants.
2	Tushi A et al, 2018, Nagaland [13]	In the tribal population of Mokokchung district, Nagaland, the prevalence of hypertension was 43.2%. The study also observed that 19.5% were current smokers and 15.9% consumed alcohol currently. A large proportion of participants reported inadequate intake of fruits and vegetables—80.1% of males and 93.6% of females. Furthermore, insufficient physical activity was noted in 3.4% of the population, and 32.4% of individuals had a BMI ranging from 23.0 to 27.49 kg/m², indicating an overweight status.
3	Ponruban C et al, 2017, Jawadhu Hills [14]	This study among the tribal population residing in Jawadhu Hills found the prevalence of hypertension to be 17.7% and diabetes mellitus to be 3.3%. Low physical activity was reported by 11.7% of the participants. The study also revealed a high prevalence of alcohol use (72.9%) and smoking (64%), indicating a high burden of behavioral risk factors among this community.
4	Sajeev P & Soman B, 2018, Thiruvanthapuram [16]	Among the Kani tribal population in southern Kerala, the prevalence of hypertension was 48.3%. The study also found that 81.5% of the participants used tobacco, while 36.2% consumed alcohol. Furthermore, inadequate physical activity was reported by 9.7% of the study participants, suggesting an emerging risk for non-communicable diseases within this tribal group.
5	Pradhan HS & Mappillairaju B, 2025, Odisha [19]	In the Santal tribes of Odisha, the study identified a hypertension prevalence of 40.3% and an obesity prevalence of 28.5%. Tobacco and alcohol use were widespread, affecting 80% and 81.8% of the population, respectively, with higher rates among males. Additionally, low physical activity was reported by 30.6%, particularly among females, and a striking 98.8% of participants had inadequate fruit and vegetable intake, reflecting unhealthy lifestyle patterns.
6	Misra PJ et al, 2014, Assam [47]	Among the Mishing tribal community of Assam, the study documented a hypertension prevalence of 26% and found that 16% were overweight. Tobacco use (84%) and alcohol consumption (67%) were highly prevalent. Moreover, an unhealthy dietary pattern was reported by 68% of the participants, emphasizing the growing burden of lifestyle-related risk factors among these tribes.
7	R K et al, 2024, Tamil Nadu [48]	In the tribal population of Tamil Nadu, the prevalence of diabetes was 7.8%. Among these individuals, 21.2% were newly diagnosed, 33.3% had diabetes for 1–5 years, 21.2% for 5–10 years, and 24.3% for more than 10 years. The study demonstrated that increasing age, higher education levels, physical inactivity, and obesity were strongly associated with the occurrence of diabetes.

## Conclusions

NCDs are one of the leading causes of morbidity and mortality among tribal populations in India, a symptom of rapid lifestyle, environmental, and socio-economic transition. Despite vigorous national efforts and the scaling up of specific public health interventions, tribal populations continue to face multidimensional obstacles, including limited access to health services, deep-rooted health inequities, lack of infrastructure, and scarcity of culturally responsive prevention and treatment services.

The evidence emphasizes that NCD risk reduction among tribal communities requires something more than scaling up services - profound integration of lenses from culture, social science, and economics into the design and delivery of interventions. Future and present interventions should prioritize community engagement highly within their agendas, facilitate local-level health workers, and incorporate NCD education and screening into tribe-specific forms. Strengthening surveillance, incentives, and training for a heterogeneous health workforce, as well as cross-sectoral policy convergence, are needed to address the complex interlinked determinants.

Above all, equity and participation should be sustained throughout. Multidisciplinary, collaborative, and context-informed strategies built on mutual trust and co-creation with tribal populations have the greatest potential to decelerate the NCD epidemic and achieve better long-term health outcomes among these underserved populations. This review identifies key health system changes and new, culturally competent interventions as foundational steps towards universal and equitable health coverage among all Indian tribes.

## References

[1] Kaur P, Borah PK, Uike PV (2022). Non-communicable diseases as a major contributor to deaths in 12 tribal districts in India. Indian J Med Res.

[2] Sathiyanarayanan S, Muthunarayanan L, Devaparthasarathy TA (2019). Changing perspectives in tribal health: rising prevalence of lifestyle diseases among tribal population in India. Indian J Community Med.

[3] (2025). World Health Organization. Global Plan of Action for the Health of Indigenous Peoples. https://www.who.int/initiatives/global-plan-of-action-for-health-of-indigenous-peoples.

[4] (2025). World Bank. Indigenous peoples overview. https://www.worldbank.org/en/topic/indigenouspeoples.

[5] Anderson I, Robson B, Connolly M (2016). Indigenous and tribal peoples’ health (The Lancet-Lowitja Institute Global Collaboration): a population study. Lancet.

[6] Ohenjo N, Willis R, Jackson D, Nettleton C, Good K, Mugarura B (2006). Health of indigenous people in Africa. Lancet.

[7] (2025). India - IWGIA - International Work Group for Indigenous Affairs. https://iwgia.org/en/india.html.

[8] Ganesh B, Rajakumar T, Acharya SK, Vasumathy S, Sowmya S, Kaur H (2021). Particularly vulnerable tribal groups of Tamil Nadu, India: a sociocultural anthropological review. Indian J Public Health.

[9] Chakma T, Kavishwar A, Sharma RK, Rao PV (2017). High prevalence of hypertension and its selected risk factors among adult tribal population in Central India. Pathog Glob Health.

[10] (2025). Icmr: Lifestyle diseases among top killers in tribal districts: ICMR | India News - Times of India. https://timesofindia.indiatimes.com/india/lifestyle-diseases-among-top-killers-in-tribal-districts-icmr/articleshow/95724990.cms.

[11] Kandpal V, Sachdeva MP, Saraswathy KN (2016). An assessment study of CVD related risk factors in a tribal population of India. BMC Public Health.

[12] Babu BV, Hazarika CR, Raina SK (2024). Prevalence of type 2 diabetes among tribal population of india: a multi-centric cross-sectional study. J Natl Med Assoc.

[13] Tushi A, Rao SR, Pattabi K, Kaur P (2018). Prevalence of risk factors for non-communicable diseases in a rural tribal population of Mokokchung, Nagaland, India. Natl Med J India.

[14] Ponruban C (2017). Prevalence of Noncommunicable Diseases and Their Risk Factors in Tribal South India - A Community Based Cross Sectional Study [PhD Dissertation]. https://1library.net/document/4zp2p60y-prevalence-communicable-diseases-factors-tribal-south-community-sectional.html.

[15] Kumar A, Bhatia M, Goel PK, Jain RB (2016). Diabetes in tribes of India: a literature review. J Soc Health Diab.

[16] Sajeev P, Soman B (2018). Prevalence of noncommunicable disease risk factors among the Kani tribe in Thiruvananthapuram district, Kerala. Indian Heart J.

[17] Shanker N, Mathur P, Das P, Sathishkumar K, Martina Shalini AJ, Chaturvedi M (2021). Cancer scenario in North-East India &amp; need for an appropriate research agenda. Indian J Med Res.

[18] Jain Y, Kataria R, Patil S (2015). Burden & pattern of illnesses among the tribal communities in central India: a report from a community health programme. Indian J Med Res.

[19] Pradhan HS, Mappillairaju B (2025). Profile of risk factors for noncommunicable diseases among the migrants of Santal tribe residing in Bhubaneswar city, Odisha, India. Natl J Community Med.

[20] Deepa K, Jose MJ, Prabhu V (2013). Prevalence and type of tobacco habits and tobacco related oral lesions among Wayanad tribes, Kerala, India. Indian J Public Health Res Dev.

[21] Verma P, Saklecha D, Kasar PK (2017). A study on prevalence of tobacco consumption in tribal district of Madhya Pradesh. Int J Med Public Health.

[22] Rose A, Mohan VR, Vinodh A, David SM, George K, Minz S, Prasad JH (2021). Hazardous use of alcohol among men in the tribal population of Jawadhi Hills, Tamil Nadu: nature, prevalence, and risk factors. J Family Med Prim Care.

[23] War RJ, Gharat VV, Chandramouleeshwaran S, Nayak S, Nimgaonkar VL, Devi S (2020). A three-site study of alcohol consumption among adolescents from indigenous tribes in India. Indian J Psychol Med.

[24] Kuhnlein HV, Receveur O (1996). Dietary change and traditional food systems of indigenous peoples. Annu Rev Nutr.

[25] Hoffman LC, Wiklund E (2006). Game and venison - meat for the modern consumer. Meat Sci.

[26] Premagowri B, Osborn J (2025). Traditional to contemporary: food consumption changes in the ruler tribal community. Biores Scientia.

[27] Crisp N (2022). Turning the World Upside Down Again: Global Health in a Time of Pandemics, Climate Change and Political Turmoil, 2nd Edition.

[28] Negi DP, Singh MM (2018). Tribal health and health care beliefs in India: a systematic review. Int J Res Soc Sci.

[29] Niazi F, Rahique A, Sriram S, Kaur KN, Saeed S (2024). Beyond numbers: decoding the gendered tapestry of non-communicable diseases in India. Int J Environ Res Public Health.

[30] Saha A, Moray KV, Devadason D (2020). Water quality, sanitation, and hygiene among the tribal community residing in Jawadhi hills, Tamilnadu: an observational study from Southern India. J Family Med Prim Care.

[31] Suresh D (2014). Tribal development through five year plans in India: an overview. Dawn J.

[32] Narain JP (2022). India at 75: transforming the health of tribal populations through evidence-based policymaking. Indian J Med Res.

[33] Kundu J, Goli S, James KS (2025). Education and non-communicable diseases in India: an exploration of gendered heterogeneous relationships. Int Health.

[34] Balarajan Y, Selvaraj S, Subramanian SV (2011). Health care and equity in India. Lancet.

[35] Setua M, Islam S (2024). Understanding the factors, impacts and management of tribal health issues: a study on Jangalmahal perspective. Int J Adv Res.

[36] Ministry of Health and Family Welfare, Government of India (2017). National Health Policy 2017. https://mohfw.gov.in/sites/default/files/9147562941489753121.pdf.

[37] Bhattacharya S, Saleem SM, Garg S, Varshney S, Grover A, Gupta P, Singh A (2024). Building a sustainable healthcare system for tribal communities through apex medical institutions. Indian J Community Med.

[38] Kumar KA, Reddy MG (2012). Political economy of tribal development: a case study of Andhra Pradesh. IOSR J Humanit Soc Sci.

[39] Ministry of Tribal Affairs, Government of India (2025). Report of the High Level Committee on Socio-Economic, Health and Educational Status of Tribal Communities of India. New Delhi: Government of India.

[40] Kumar MM, Pathak VK, Ruikar M (2020). Tribal population in India: a public health challenge and road to future. J Family Med Prim Care.

[41] Ministry of Health and Family Welfare, Government of India (2013). National Urban Health Mission: Framework for Implementation. https://nhm.gov.in/images/pdf/NUHM/Implementation_Framework_NUHM.pdf.

[42] Bhatia V, Behera P (2017). Tribal health care: the unaddressed aspect in the Indian health system. Indian J Community Fam Med.

[43] Srikanth N (2021). Integrative medicine in india: need for an inclusive health policy. Trad Med Rev.

[44] (2025). Press Information Bureau, Government of India. Tribal health mission launched in Chhattisgarh. https://www.pib.gov.in/PressReleasePage.aspx?PRID=2061196.

[45] (2025). Press Information Bureau, Government of India. Cabinet approves Pradhan Mantri Janjatiya Unnat Gram Abhiyan. https://www.pib.gov.in/PressReleasePage.aspx?PRID=2055996.

[46] Tangcharoensathien V, Mills A, Das MB, Patcharanarumol W, Buntan M, Johns J (2018). Addressing the health of vulnerable populations: social inclusion and universal health coverage. J Glob Health.

[47] Misra PJ, Mini GK, Thankappan KR (2014). Risk factor profile for non-communicable diseases among Mishing tribes in Assam, India: results from a WHO STEPs survey. Indian J Med Res.

[48] R K, Sundaram P, M L, Vv A, Leon N (2024). Prevalence of diabetes and its associated factors among the adult tribal population in Tamilnadu, India. Natl J Community Med.

